# Digital Implementation of Oscillatory Neural Network for Image Recognition Applications

**DOI:** 10.3389/fnins.2021.713054

**Published:** 2021-08-26

**Authors:** Madeleine Abernot, Thierry Gil, Manuel Jiménez, Juan Núñez, María J. Avellido, Bernabé Linares-Barranco, Théophile Gonos, Tanguy Hardelin, Aida Todri-Sanial

**Affiliations:** ^1^Laboratoire d'Informatique, de Robotique et de Microélectronique de Montpellier, University of Montpellier, CNRS, Montpellier, France; ^2^Instituto de Microelectronica de Sevilla, IMSE-CNM, CSIC, Universidad de Sevilla, Sevilla, Spain; ^3^A.I. Mergence, Paris, France

**Keywords:** artificial intelligence, auto-associative memory, FPGA implementations, learning rules, oscillatory neural networks, pattern recognition

## Abstract

Computing paradigm based on von Neuman architectures cannot keep up with the ever-increasing data growth (also called “data deluge gap”). This has resulted in investigating novel computing paradigms and design approaches at all levels from materials to system-level implementations and applications. An alternative computing approach based on artificial neural networks uses oscillators to compute or Oscillatory Neural Networks (ONNs). ONNs can perform computations efficiently and can be used to build a more extensive neuromorphic system. Here, we address a fundamental problem: can we efficiently perform artificial intelligence applications with ONNs? We present a digital ONN implementation to show a proof-of-concept of the ONN approach of “computing-in-phase” for pattern recognition applications. To the best of our knowledge, this is the first attempt to implement an FPGA-based fully-digital ONN. We report ONN accuracy, training, inference, memory capacity, operating frequency, hardware resources based on simulations and implementations of 5 × 3 and 10 × 6 ONNs. We present the digital ONN implementation on FPGA for pattern recognition applications such as performing digits recognition from a camera stream. We discuss practical challenges and future directions in implementing digital ONN.

## 1. Introduction

In recent years, we have witnessed a proliferation of smart edge devices adopted by all industry sectors such as smart homes, smart city cameras, smart autonomous driving cars, smart healthcare, smart manufacturing, etc. Most edge devices are getting smaller and compact.

Using Artificial Neural Networks (ANNs), specifically Deep Neural Networks (DNNs) to create Artificial Intelligence (AI) at the edge, has successfully been used to teach smart systems to recognize or detect objects (Redmon et al., 2016; Krizhevsky et al., 2017; Shah and Kapdi, 2017; Yang and Song, 2018; Jiao et al., 2019), read texts (Jackel et al., 1991), and understand speeches (Xiong et al., 2018; Nassif et al., 2019). Contraints to such applications on edge devices derives from the inherent limitations of power consumption, memory, and little to no bandwidth. In addition, privacy and security concerns would recommend the data to be stored locally. In contrast, ANNs or DNNs systems that enable AI at the edge are getting larger to cope with the ever-increasing amount of data. Thus, resulting in more power consumption, memory, and bandwidth demand.

Systems based on ANNs and Convolutional Neural Networks (CNNs) running on traditional von Neumann architectures require a large amount of memory, computational power, and bandwidth demand. While they perform well on expensive hardware such as GPUs (Pham et al., 2019), they are often unsuitable for smaller edge devices. Such a disconnect between the growing need in AI at the edge and limitations of processing hardware has compelled significant research efforts into beyond-von Neumann systems such as neuromorphic computing paradigms deployable at the edge (Bey, 2020; Kendall and Kumar, 2020).

This paper focuses on an alternative, low-power, neuromorphic computing approach with Oscillatory Neural Networks (ONNs) (Raychowdhury et al., 2019; Csaba and Porod, 2020). The ONN is a system of coupled oscillators mimicking at circuit level the basic structure of the brain architecture. The key feature of ONNs is to encode the information on the phase relations between oscillators and to let them oscillate using their physical dynamics to compute. For example, the random start of five metronomes (similar to grandfather's clock) will make them oscillate in parallel (Met, 2013). After several cycles, they get synchronized in frequency while their phase relations can tell us if they are in- or out-of-phase. Contrarily to the classical computation based on voltage amplitude to determine a logic “1,” or “0,” in ONN we use the phase relations to determine the logic “1” (out-of-phase 180^*o*^) or “0” (in-phase 0^*o*^). Thus, working with parallel oscillators in the frequency and phase domains allows to reach fast and low-power computation (Roychowdhury, 2014; Shukla et al., 2016). This makes ONN an ideal solution to bring artificial intelligence on edge devices.

ONN principle was first introduced in Hoppensteadt and Izhikevich (2000) where ONN showed good associative memory properties. Thus, there is a recent interest to exploit ONN for large-scale associative memory applications. While there is a lot of ongoing research on devices and analog architectures to implement ONN efficiently (Csaba and Porod, 2013; Jackson et al., 2015; Shi et al., 2016; Kumar and Mohanty, 2017; Corti et al., 2019; Velichko et al., 2019), we focus on addressing a more fundamental problem—can we perform relevant AI applications such as image recognition with ONN? We explore ONN at small-scale (up to 60 coupled oscillators) as a first attempt to show phase computation in the digital domain. Despite being a small-scale ONN, we investigate advantages and limitations on image recognition tasks suitable for AI applications on edge devices. To do so, we implement an FPGA-based digital ONN to serve as a proof-of-concept of the ONN computing paradigm for enabling AI at the edge.

The rest of the paper is organized as follows. In section 2, we present materials and methods used for all experiments carried out for this work. In section 2.1, we introduce the ONN model and compare it with state-of-the-art associative memory models. Then, in section 2.2, we present the training methods we apply to the ONN. Afterward, in section 2.3, we describe the digital ONN design. section 2.4 presents methods used for ONN validation and characterization for design simulation and FPGA implementation. Next, section 2.5 exposes methods for a 10 × 6 ONN used for image recognition from a camera stream. section 3 reports on results related to ONN simulation, ONN FPGA implementation, and image recognition application using such 10 × 6 ONN. Finally, in section 4, we discuss the advantages and limits of ONN and future directions.

## 2. Materials and Methods

### 2.1. ONN Biological Inspiration and Related Works

Recent researches on brain-inspired computing paradigms have focused on Spiking Neural Networks (SNNs) (Maass, 1997; Paugam-Moisy and Bohte, 2012; Zenke and Ganguli, 2018). Neurons in the brain use voltage spikes to transmit information and SNNs emulate spikes to compute efficiently in the time domain. On another side, the human brain's electrical activity has shown macroscopic oscillations observable on electroencephalogram signals (EEG). Since then, on going research has tried to find relationships between oscillation behavior and neuronal activity (Martindale, 1978; Lieff, 2012). ONNs are a novel computing paradigm that uses coupled oscillators as neurons to mimic brain wave oscillations. With ONNs, we exploit the synchronization dynamics of physical oscillators to compute (Hoppensteadt and Izhikevich, 1997).

In ONNs, information is encoded in the phase of the oscillators. Selecting one oscillator as the reference, we can use each oscillator to encode frame values into phases (between 0° and 180°). Computation consists of oscillators initialization with initial phase state ϕ_*init*_ ($0^{o}<\phi_{init}<180^{o}$). Then, oscillators will oscillate in parallel within multiple states until they stabilize and lock in phase. Once they stabilize, we can measure phase information of the final state ϕ_*end*_ and associate it to output frame values. When synchronization occurs, the oscillators oscillates in parallel so it permits fast computation, independently from the number of oscillators. Additionally, computing with oscillators in the frequency domain allows low voltage operation. These two features allow for low power computation (Roychowdhury, 2014; Shukla et al., 2016), and are attractive to implement artificial intelligence on edge devices.

Thus, ONN advantages come from the analog-based computing principle and the hardware implementation. Different approaches have been explored to emulate oscillating neurons in ONN architectures such as using spin-torque oscillators (Csaba and Porod, 2013), ring oscillators (Csaba et al., 2016), microelectromechanical oscillators (Kumar and Mohanty, 2017), PLL-based oscillators (Shi et al., 2016), or more recently, beyond-CMOS devices such as *VO*_2_-based oscillators (Corti et al., 2019). In parallel, different analog couplings have been proposed using resistors and capacitors (Csaba and Porod, 2020). In literature, (Ahmed et al., 2021) reported a fully coupled ONN implementation of 100 neurons. It is, to the best of our knowledge, the largest implementation of an ONN with fully coupled oscillators. Other efforts have been deployed on the hardware implementation of ANNs (Misra and Saha, 2010; Levi et al., 2018) and SNNs have shown a particular interest for hardware implementations due to their low-power properties. Some authors have developed FPGA-based platforms with SNNs (Rosado-Muñoz et al., 2012; Guo et al., 2021), and mostly perform image classification tasks (Han et al., 2020; Xia et al., 2020). Some others have focused more on customizable neuromorphic chips (Khan et al., 2008; Mitra S, 2009; Ma et al., 2017; Davies et al., 2018). In this manuscript, we present a first proof-of-concept of the ONN paradigm implemented on FPGA, and further efforts are needed to develop ONN-based hardware implementations at the same scale as existing SNN neuromorphic chips.

**Figure 1:**
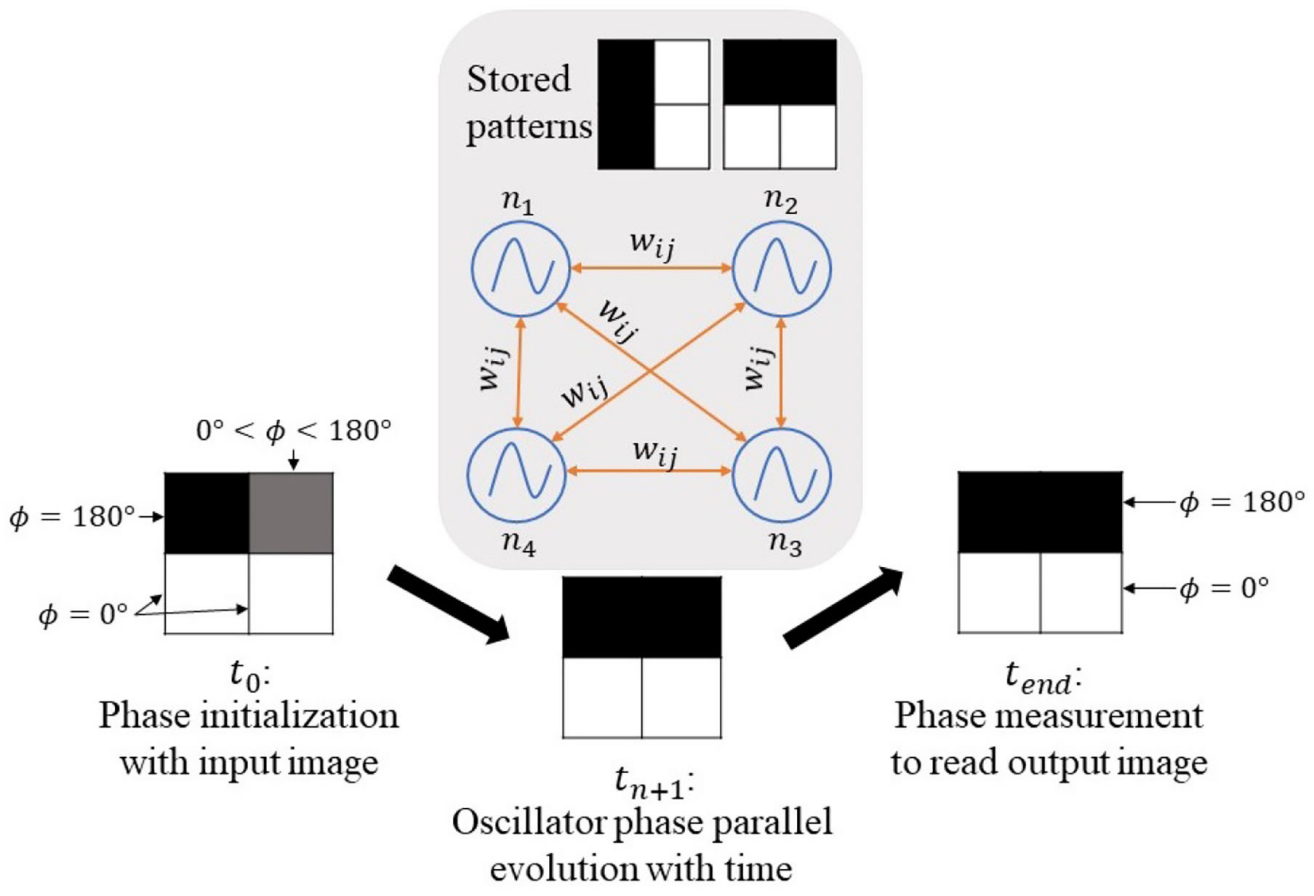
Oscillatory Neural Network with 4 neurons *n* using associative memory capability to perform image recognition (*w*_*ij*_ corresponds to the weight between neurons *n*_*i*_ and *n*_*j*_).

ONNs have shown associative memory computing properties (Hoppensteadt and Izhikevich, 2000), like the one possessed by Hopfield Neural Networks (HNNs) (Hopfield, 1982). Associative memory systems can store patterns and associate each possible input to one of the stored patterns. Stored patterns represent the minima of an energy function toward which the network evolves. In the case of multiple stored patterns, the network will evolve and converge to the nearest energy minimum, meaning the closest stored pattern from the input. We train the ONN to store patterns by adapting the coupling between oscillators. The coupling elements represent the weights of the oscillators. If we consider the image processing domain, each pixel is an oscillator, and the phase information represents the pixel color. We compute weights corresponding to training images stored in ONN. Thus, when we initialize the network with a new image, it converges to the closest stored image, see Figure 1. This is what we define by image recognition task in this paper. In some cases, the network evolves between states without stabilizing, meaning the network does not converge.

HNNs have shown capabilities to work with binary/bipolar-valued patterns (Hopfield, 1982), continuous-valued patterns (Ramsauer et al., 2021), and complex-valued patterns (Muezzinoglu et al., 2003; Tanaka and Aihara, 2009). In this work, we use binary patterns representing in- and out-of-phase relations and corresponding to black and white images.

### 2.2. ONN Learning

The Hebbian learning rule (Morris, 1999) is one of the most popular learning algorithm to calculate synaptic weights for bipolar-valued stored patterns on HNNs. However, the Storkey learning rule (Storkey et al., 1997) has been reported to possess higher storage capacity and robustness against correlated stored patterns and crosstalk phenomenon (Storkey, 1997; Wu et al., 2012). So, we apply both the Hebbian and the Storkey rules to the ONN and compare results.

For both learning rules, we transform each stored pattern with index *k* into a vector ξ^*k*^ of length *N*, with *N*, the number of neurons inside the network. Each vector element is bipolar (–1/1). For the Hebbian rule, synaptic weight *w*_*ij*_ between neuron *n*_*i*_ and neuron *n*_*j*_ is calculated as:

(1) $$\begin{array}{l} w_{ij}=\underset{k}{\sum }\xi_{i}^{k}\xi_{j}^{k^{T}} \end{array}$$

with *w*_*ij*_ = 0 ∀ *i* = *j*.

Whereas, Storkey learning rule is defined as:

(2) $$\begin{array}{l} w_{ij}^{\nu }=w_{ij}^{\nu -1}+\frac{1}{N}\xi_{i}^{\nu }\xi_{j}^{\nu }-\frac{1}{N}\xi_{i}^{\nu }h_{ji}^{\nu }-\frac{1}{N}h_{ij}^{\nu }\xi_{j}^{\nu } \end{array}$$

(3) $$\begin{array}{l} h_{ij}^{\mu }=\sum_{k=1,k\neq i,j}^{N}w_{ik}^{\mu -1}\xi_{k}^{\mu } \end{array}$$

where $w_{ij}^{0}=0$ ∀ *i, j*, and *h*_*ij*_ is a form of local field at neuron *i*.

Theoretically, the Hebbian memory capacity, represented by the maximum number of stored patterns *K* is derived in Amit et al. (1987) as

(4) $$\begin{array}{l} K=0,14*N \end{array}$$

For our simulations and implementations, we apply up to *K* patterns. We calculate weights off-line using a software algorithm and we store them in our digital design.

### 2.3. Digital ONN Design

We develop a digital ONN as a proof of concept of the computing in phase paradigm to explore ONN architectures and AI at-the-edge applications. We present an ONN digital design inspired by hybrid analog-digital work from Jackson et al. (2019) but without analog components.

**Figure 2:**
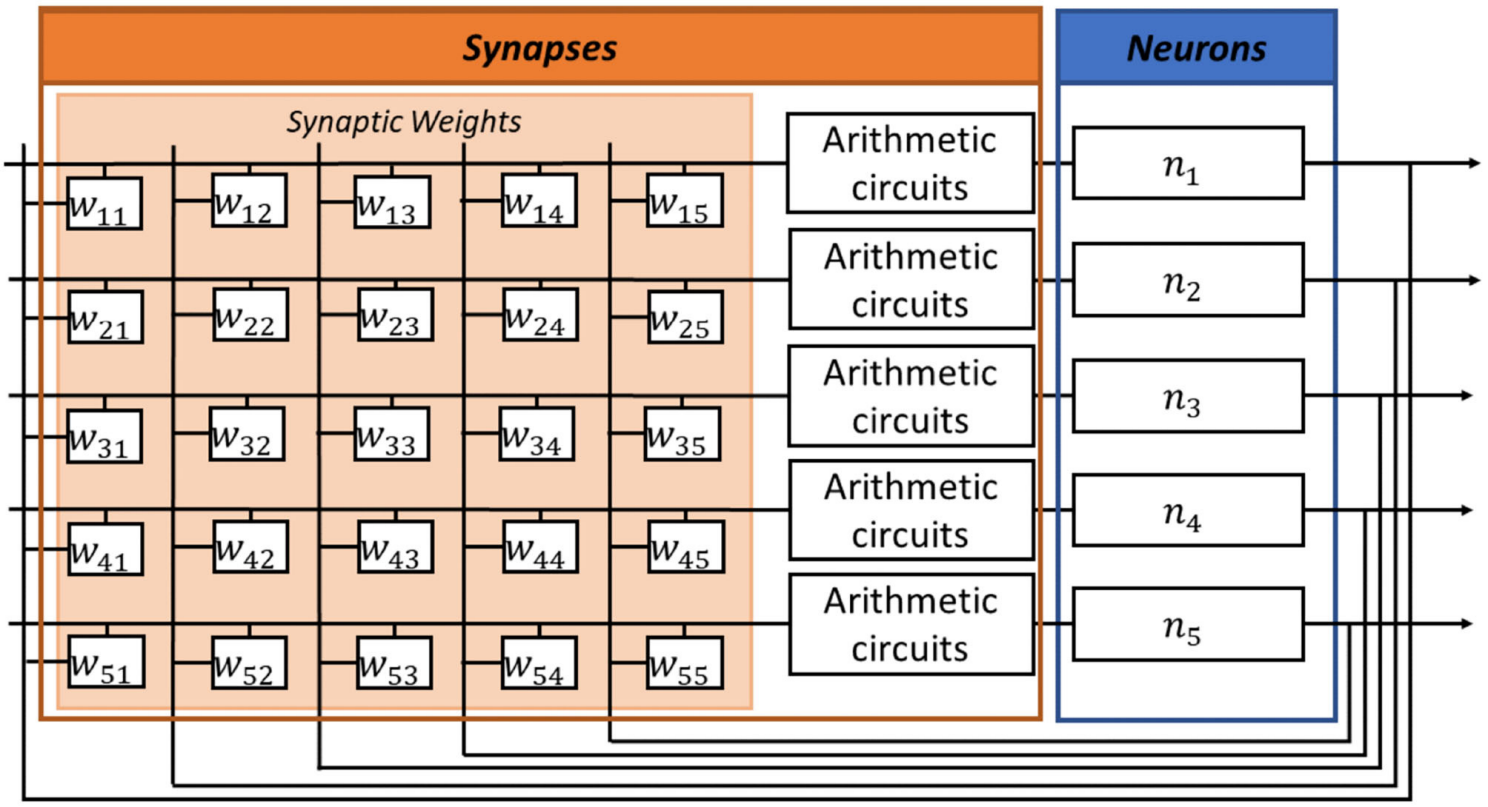
Digital ONN architecture with fully parallel implementation of neurons and a combinatorial implementation of the arithmetic synapse block.

**Figure 3:**
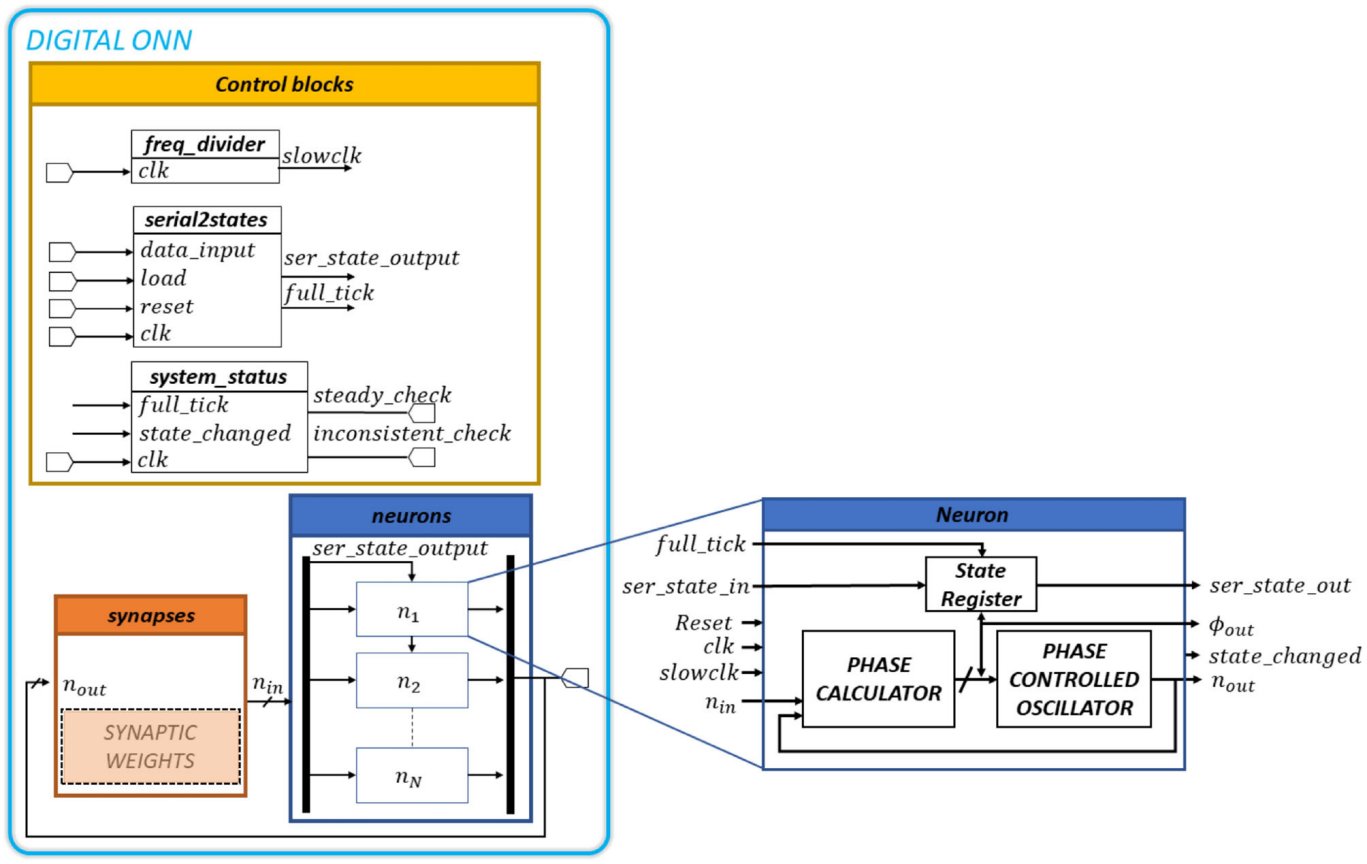
Complete digital ONN design representing digital blocks and block description with the operating principle of a single oscillatory neuron.

In Jackson et al. (2019), synapses are implemented by a resistor network, and a critical analog comparator is required at the input of each digital neuron. In contrast, in our digital ONN implementation, we use an arithmetic circuit for each synapse and there is no analog comparator in neurons. Figure 2 illustrates the digital ONN architecture. It is a fully parallel design, meaning we implement each neuron inside the FPGA to oscillate in parallel. In addition, the synapses block is combinatorial, which means all synaptic operations are computed in parallel. Also, our architecture needs extra blocks to control synapses and neuron signals. Figure 3 presents our digital ONN design composed of neurons, synapses, and control blocks. In the following subsections, we detail the implementation of each block.

#### 2.3.1. Neuron Block

In this design, ONN neurons are phase-changed oscillators. Each neuron *n* computes the phase difference between the present input and output oscillations to align the output in-phase with the input. All neurons are identical according to the diagram in Figure 3. A neuron has one 1-bit input, *n*_*in*_, and one 1-bit output, *n*_*out*_, oscillating signals (square signals), in addition to synchronization, initialization, and control signals. The synapse block generates the *n*_*in*_ signal, which determines the evolution of the neuron phase. The ϕ_*out*_ and *state*_*changed* signals give information on the neuron phase and its evolution. We use *full*_*tick*, *ser*_*state*_*in*, and *ser*_*state*_*out* signals to initialize the neuron phase, and *reset*, *clk*, and *slowclock* signals to ensure synchronization.

The process starts with the initialization of the output signal phase ϕ_*out*_. It triggers the initial output oscillation *n*_*out*_ corresponding to ϕ_*out*_. Automatically, the synapses block computes the new input oscillation *n*_*in*_ with the phase ϕ_*in*_. We initialize all neurons serially when *full*_*tick* is activated by connecting *ser*_*state*_*out* signal to *ser*_*state*_*in* signal of the neighbor neuron. Note, when initialization is over, a scan-path between *ser*_*state*_*out* and *ser*_*state*_*in* is configured to load and read ONN's state in series.

Then, each neuron calculates the phase difference Δϕ between ϕ_*in*_ and ϕ_*out*_ and uses it to update the new ϕ_*out*_ with a phase calculator block. The phase calculator block contains two edge detectors and a finite state machine (FSM). Edge detectors detect rising edges on *n*_*in*_ and *n*_*out*_ oscillating signals. FSM measures the time difference between *n*_*in*_'s rising edge and *n*_*out*_'s rising edge to define Δϕ. The Δϕ value allows us to update the neuron output phase ϕ_*out*_ aligning *n*_*out*_ signal with *n*_*in*_ signal, as:

(5) $$\begin{array}{l} \phi_{out}=\phi_{out}+/-\Delta \phi \end{array}$$

Note, the sign (+/−) depends on the first rising edge detected. (−) if *n*_*out*_'s rising edge is detected first and (+) if *n*_*in*_'s rising edge is detected first. Note, the *n*_*in*_ signal phase is set by the weighted sum of the neuron's input signals.

**Figure 4:**
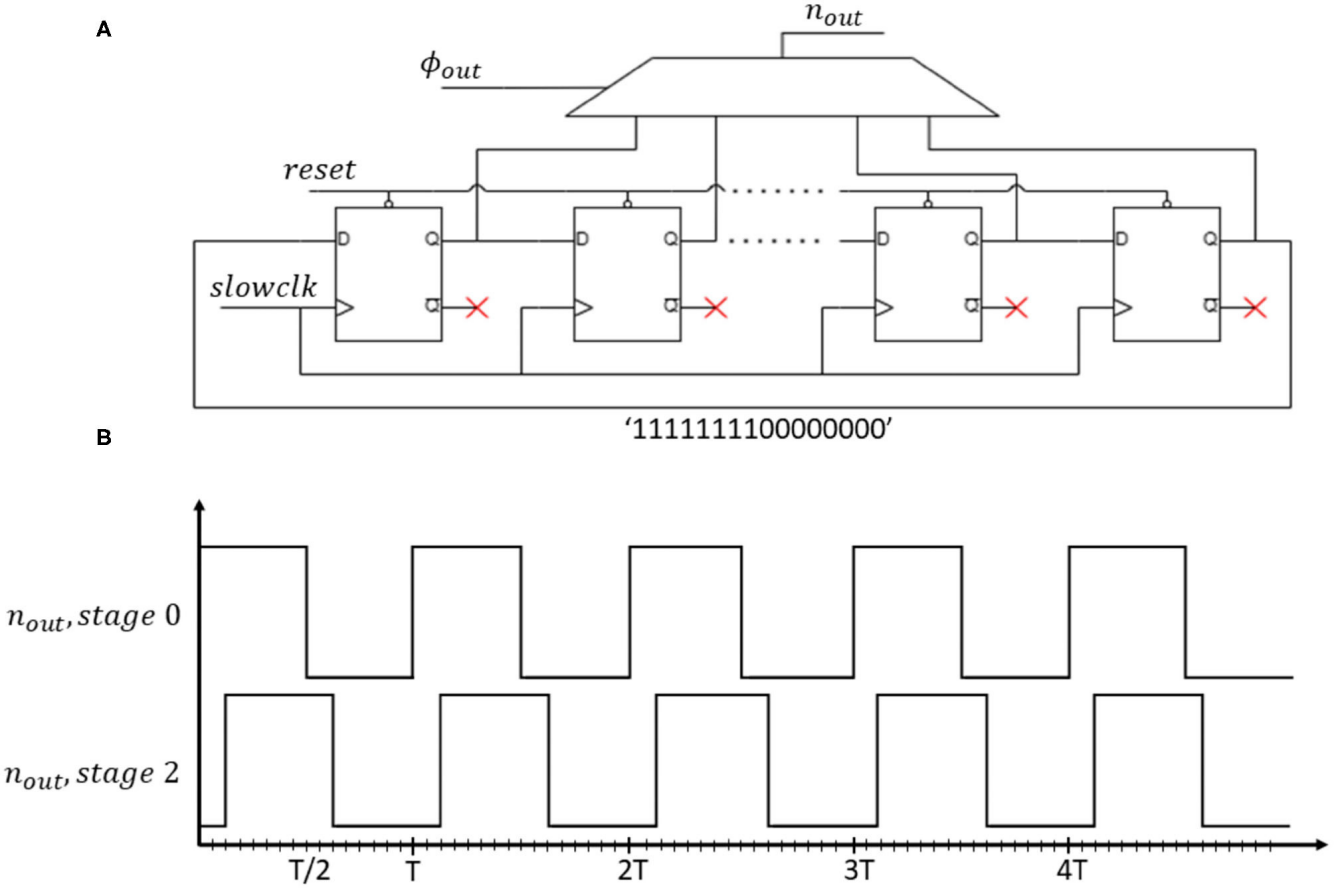
**(A)** Logic diagram of the implemented phase-controlled oscillator. **(B)** Output waveform of internal neuron shift-register for stages 0 and 2.

Finally, we apply the new ϕ_*out*_ to the oscillating output signal *n*_*out*_ with a phase-controlled oscillator. The phase-controlled oscillator contains a circular shift register with a multiplexer. The shift register has 16 stages to represent square signals with different phases so, 16 phase options are available. The 16-bit pattern [1111111100000000] cycles continuously through time. So, through the multiplexer selection bits, we select a shift-register state corresponding to a square signal with a distinct phase. This square signal becomes the new neuron output *n*_*out*_. Figure 4A shows the logic diagram of the phase-controlled oscillator and Figure 4B the waveforms corresponding to stage 0 (in-phase, ϕ_*out*_ = 0°) and stage 2 (out-of-phase, ϕ_*out*_ = 45°). The register controlling the multiplexer stores the neuron state, or equivalently the phase of the neuron output. Note, we use different clocks (driven by the system clock) to control the state register and the shift register. The latter is driven by a slow clock generated from the system clock so that the multiplexer's output cycles as long as its control register remains unchanged with a period *T*_*osc*_ = 16 * *T*_*slowclk*_.

#### 2.3.2. Synapses Block

ONN synapses block contains weights and computes each neuron input oscillation *n*_*in*_. We use an arithmetic logic circuit for our digital ONN design to generate the input signal to the *i*-th neuron as:

(6) $$\begin{array}{l} n_{in}\left[i\right]=sign\left(\underset{j}{\sum }w_{ij}-\underset{k}{\sum }w_{ik}\right) \end{array}$$

where *j* extends to those neurons with *n*_*out*_[*j*] = 1 and *k* to those with *n*_*out*_[*k*] = 0. It is the most expensive component in terms of resources. Results in this paper correspond to a combinatorial synapses block using 5-bit weights.

#### 2.3.3. Control Block

In addition to neurons and synapses, our digital design requires a control block to control and monitor ONN computation. It is mainly in charge of three tasks. (1) The initialization step required to carry out an ONN computation. We serially apply input state with a scan-path on neuron's state registers while activating the *full*_*tick* signal. (2) The control block generates a slow clock to ensure ONN operation. The relation between the slow clock and the system clock is *T*_*slowclk*_ = 4 * *T*_*clk*_. We use a frequency divider of 2-bit length to speed up the system performance. (3) The generation of the steady (*steady*_*check*) and the inconsistent (*inconsistent*_*check*) signals (see Figure 3). They indicate wether ONN gives a stable or unstable state. We activate the steady signal once the ONN reaches a stable state, meaning all neuron phases ϕ_*out*_ are stable for two oscillation periods (*T*_*osc*_). We activate as well the inconsistent signal if the ONN does not achieve any stable state after a time (160 μ*s*) arbitrarily defined by us. To do so, the control block monitors neurons' oscillation activity.

The combination of neuron blocks, synapses block, and control block creates our complete fully-digital ONN design. Next, we carry out tests to validate the associative memory properties of our design, and its characteristics.

### 2.4. ONN Characterization Methods

To validate our digital ONN design, we characterize 5 × 3 and 10 × 6 digital ONNs using both Hebbian and Storkey learning rules. First, in section 2.4.1, we present simulation methods used to evaluate its performances using software tools. Next, in section 2.4.2, we present FPGA implementation methods.

#### 2.4.1. Simulation

We validate and characterized ONN with simulation software tools using a testbench before being implemented on FPGA. We use a testbench on the software Xilinx's Vivado Design Suite 2018.2. We also perform post-place&route simulations to characterize ONN designs following Vivado's default strategies for synthesis and implementation. We set the target device to the Xilinx 7-series FPGA, the XC7Z020-1CLG400C since it is used for implementation afterward.

**Figure 5:**
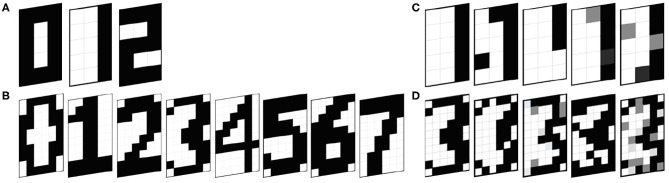
Reference patterns for **(A)** 5 × 3 ONN, **(B)** 10 × 6 ONN, and Test sets **(C)** associated to digit 1 for the 5 × 3 ONN, **(D)** associated to digit 3 for the 10 × 6 ONN.

We carry out simulations with a 5 × 3 ONN and a 10 × 6 ONN configured for pattern recognition using both Hebbian and Storkey learning rules. We configure the 5 × 3 ONN design with three stored patterns with standard 5 × 3 bitmap representations of digits 0, 1, and 2 (see Figure 5A). Each pixel of the image corresponds to a neuron. Each pixel color is associated with each neuron phase, with white as in-phase (0°), and black as out-of-phase (180°). Gray-level pixels are encoded with intermediate phases. Similarly, we configure the 10 × 6 ONN design with five stored patterns representing digits 0, 1, 2, 3, and 4 (see Figure 5B). We use two test sets, one for each ONN, containing both stored patterns and corrupted patterns. We create four corrupted patterns associated with each stored pattern by changing several pixel values with opposite or intermediate values (black or white or gray), see Figures 5C,D. We use Hamming Distance (HD) as a metric to measure the corrupted patterns' deviation from the stored ones. The HD between two patterns ξ^ν^ and ξ^μ^ of *i* elements is defined as:

(7) $$\begin{array}{l} HD=\frac{1}{2}\underset{i}{\sum }\left(\xi_{i}^{\nu }-\xi_{i}^{\mu }\right) \end{array}$$

In our test set, each stored pattern has four associated corrupted ones that are closer in their Hamming distances than any other stored patterns. Corrupted patterns are supposed to stabilize on the stored pattern with closer HD.

#### 2.4.2. FPGA Implementation

Once we validated and characterized the ONN design using simulation, we implement it on an FPGA to ensure ONN operation on a real embedded platform and to measure real performances. Here, we describe the experimental set-up necessary for ONN implementation on FPGA.

We test real pattern recognition performances of 5 × 3 and 10 × 6 digital ONN designs by implementing them on an FPGA chip. We choose to use a Zybo-Z7 Digilent development board (Digilent, 2018). The board has many communication ports, memory spaces, user interaction tools, and a Xilinx Zynq-7000 System on Chip (SoC). The SoC integrates a dual-core ARM Cortex-A9 processor with Xilinx 7-series FPGA, the XC7Z020-1CLG400C. Only FPGA resources are necessary for the digital ONN implementation. As for simulation, we use Xilinx's Vivado Design Suite 2018.2 software to implement the digital ONN design on FPGA.

**Figure 6:**
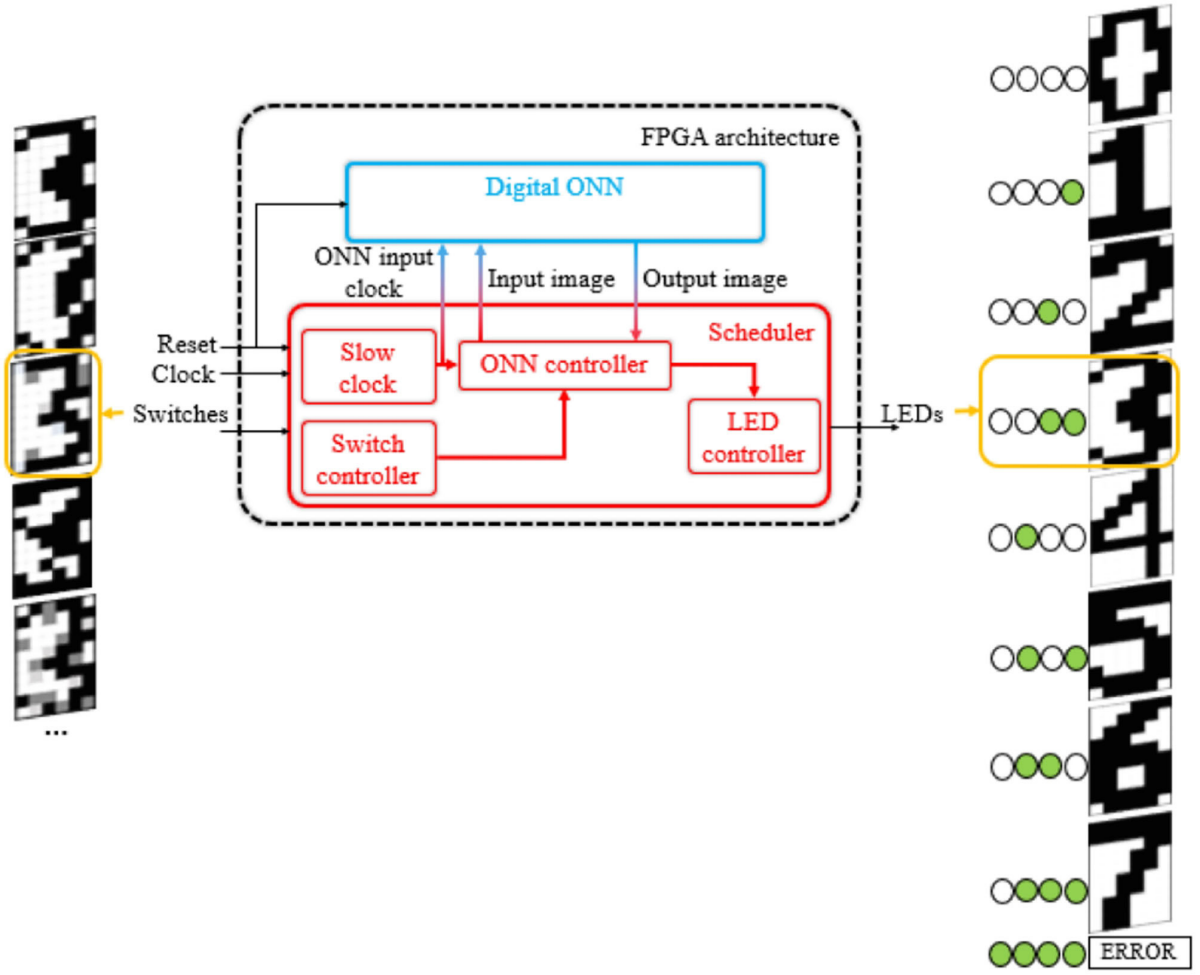
ONN FPGA implementation architecture for pattern recognition on Zybo-Z7 development board.

Figure 6 shows the system level architecture, including the digital ONN design, for performing pattern recognition on FPGA. The architecture includes the digital ONN described in section 2.3 and a scheduler block to control it. The scheduler has four control blocks to monitor and check the ONN operations. First, the system clock is divided inside the slow clock block to ensure the operations. Test patterns are stored inside the ONN controller and we use switches to select the input pattern. Next, the controller sends the input pattern to the ONN and waits until the end of ONN computation (steady signal activated). In the end, the ONN controller measures the ONN output state, applies a mask to identify the stored image, and the LED controller block turns *on*/*off* the corresponding LEDs, indicating which stored image ONN retrieves. The development board provides switches and LEDs needed by the architecture.

First, we validate our ONN FPGA implementation by performing the same tests as in simulation, and we compare results. We define a training configuration with two parameters; the learning rule and the stored pattern combination. Respecting the Hebbian maximum capacity (stored pattern limit) described in section 2.2, we try multiple stored pattern combinations for both Hebbian and Storkey learning rules for the 5 × 3 ONN and the 10 × 6 ONN, see Table 1. Similar to simulation characterization, the test set includes stored patterns plus four corrupted versions of each. See examples on Figure 5.

**Table 1:** Pattern combinations used for implementation characterization.

**ONN size**	**Number of stored patterns**	**Patterns**
5 × 3	2	0, 1
5 × 3	2	0, 2
5 × 3	2	1, 2
5 × 3	3	0, 1, 2
10 × 6	3	0, 1, 2
10 × 6	4	0, 1, 2, 3
10 × 6	5	0, 1, 2, 3, 4
10 × 6	6	0, 1, 2, 3, 4, 5
10 × 6	7	0, 1, 2, 3, 4, 5, 6
10 × 6	8	0, 1, 2, 3, 4, 5, 6, 7

We also test multiple frequencies to compare with the maximum frequency evaluated in simulation. ONN input frequency is defined by the system clock of the development board (125 MHz) divided by a configurable factor. The configurable factor allows us to divide the frequency by multiples of 2. First, we set the ONN input frequency to 7.8125 MHz which is much lower than the frequency estimated by simulation static timing analysis. Then, we modify the configurable factor to try higher frequencies, up to the maximum frequency 125 MHz.

We use the error rate (*E*_*R*_) metric to check the ONN operation. It is computed as:

(8) $$\begin{array}{l} E_{R}=\frac{\epsilon }{I_{tests}} \end{array}$$

with ϵ, the number of errors and *I*_*tests*_, the number of test images. We consider an output as an error when the retrieved pattern does not correspond to any stored ones or when the ONN does not stabilize (inconsistent signal activated).

Next, we experimentally measure initialization time (*t*_*init*_), and computation time (*t*_*comp*_) of the ONN with an oscilloscope to calculate the number of frames per second (*FPS*) that ONN implemented on FPGA can treat. It is calculated as:

(9) $$\begin{array}{l} FPS=\frac{1}{t_{init}+t_{comp}} \end{array}$$

where initialization is the time needed to send serially the test pattern to the ONN. Thus, initialization time grows linearly with the increase of the ONN size.

### 2.5. Digits Recognition Application Methods

To prove the ONN's capability to perform real-world applications, a 10 × 6 ONN is implemented on FPGA inside a complete image recognition system with a camera stream.

**Figure 7:**
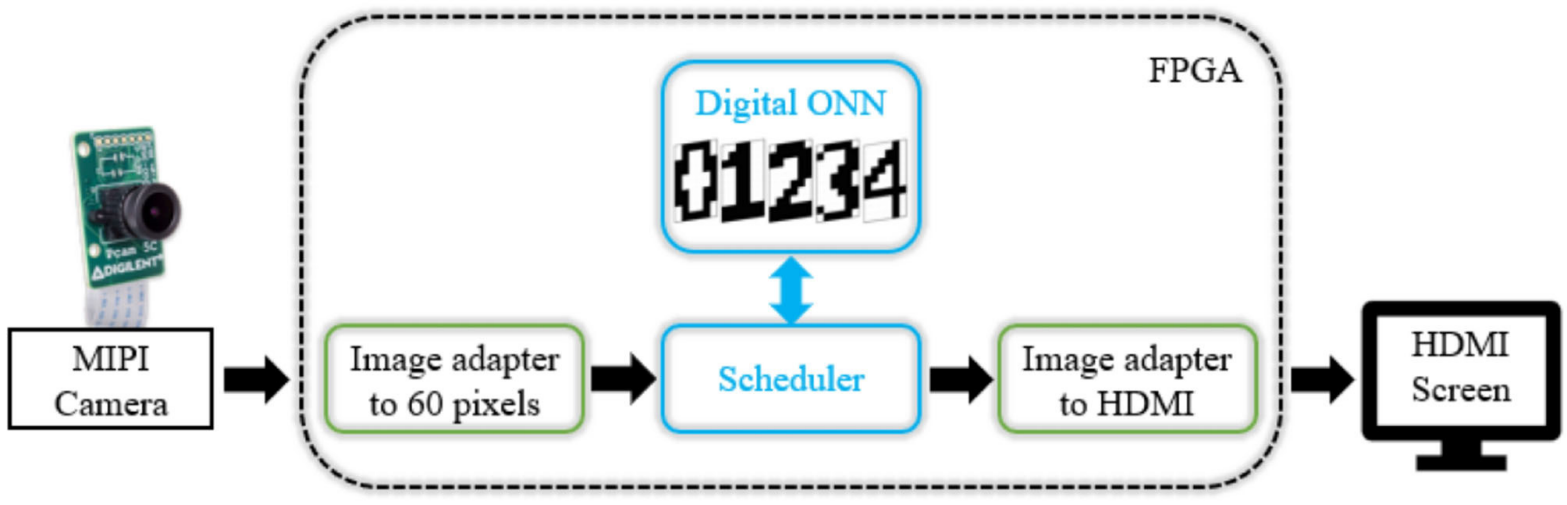
System-level architecture for image recognition application.

We configure a 10 × 6 ONN to recognize digits. We use a camera to stream digits as the input image for ONN. Input images are displayed by a phone to the camera with a dedicated application. The camera is connected to the development board and sends input images to the ONN. When the computation time is over, the output pattern is displayed on an external screen (see Figure 7).

We use a Pcam 5c (Digilent, 2017) camera which is connected via a MIPI CSI-2 (MIPI Camera Serial Interface 2) with the Zybo-Z7 development board. We connect the external screen via HDMI (High Definition Multimedia Interface) communication. The image streaming from the camera to the screen comes from a Digilent Github project named Zybo-Z7-Pcam-5c (Digilent, 2020), compatible with the Xilinx's Vivado software 2018.2. We embed the digital ONN inside the image treatment flow. To do so, we convert the camera's image in greyscale, binarize it in black/white pixels, and scale it down to a 10 × 6 pixels image taking the primary color between black/white pixels. We rescale ONN output into a 1280x720 pixels image to display it on the screen. Both rescaling steps use the Vivado HLS tool from Xilinx. We also use development board processor resources to configure the camera. Based on our characterization results, we can parametrize the ONN to a certain training configuration. We choose to train the 10 × 6 ONN to recognize five digits, from 0 to 4, with the Hebbian learning rule. The test set comprises five trained images and 20 corrupted images, similar to ONN characterization, so we expect equal results.

## 3. Results

### 3.1. Characterization Results

We validate and characterize digital ONN design with pattern recognition configurations described in section 2.4 and analyze ONN performances. We characterize 5 × 3 and 10 × 6 digital ONNs using both Hebbian and Storkey learning rules. First, in section 3.1.1, we show simulation results and ONN performances. Then, in section 3.1.2, we detail results obtained with the ONN FPGA implementation.

#### 3.1.1. Simulation

We simulate 5 × 3 and 10 × 6 ONNs and report on pattern retrieval for Hebbian and Storkey learning rules. Simulation tests of the 5 × 3 ONN using Hebbian and Storkey learning rules result in only 1 test pattern not correctly retrieved. In contrast, simulation tests of the 10 × 6 ONN have different results with the Hebbian learning rule or the Storkey learning rule. Using Hebbian, 5 test images out of 25 do not converge precisely to their respective stored pattern. However, using Storkey, the ONN design retrieves the whole test set successfully. It confirms Storkey's better storage capacity mentioned in section 2.2. We achieve pattern recognition task, with a worst case of 5 images not retrieved (errors) out of 25 (20% error rate).

Additional post place&route simulation allows us to extract estimated characteristics about maximum operating frequency and required resources, see Table 2. Such results indicate that both frequency and logical resources are highly dependent on the number of neurons. The smaller the network size is, the higher the system clock frequency can be. Note that frequency differs for the 10 × 6 ONN design that uses Hebbian or Storkey learning rules. Frequency is limited by the synapses block, and synaptic weights are different when using one or another learning rule. It changes the critical path length leading to different frequency limits. In any case, note that differences are almost insignificant.

**Table 2:** Frequency limit and resource utilization estimated in simulation for Xilinx 7-series FPGA.

**Design**	**Maximum**	**LUTs**	**Flip-Flops**
	**frequency**	**(%)**	**(%)**
5 × 3 - Hebbian	83.33 MHz	958 (1.8)	721 (0.68)
5 × 3 - Storkey	83.33 MHz	800 (1.5)	721 (0.68)
10 × 6 - Hebbian	64.10 MHz	6,426 (12.08)	2,756 (2.59)
10 × 6 - Storkey	60.61 MHz	6,192 (11.64)	2,756 (2.59)

Besides, the 5 × 3 ONN design requires nearly ten times fewer resources than the 10 × 6 ONN design. It highlights one of the digital ONN design limits. An increase in the ONN size extends ONN logical resources. Depending on the used FPGA, the number of neurons will be limited. In the next experiments, we use a Xilinx-7 series FPGA. Table 3 details resource utilization for multiple ONN sizes on the Xilinx 7-series FPGA. It shows the increase in LUTs with ONN size. For the given ONN design, we can implement stand-alone ONNs between 140 and 150 neurons.

**Table 3:** Resource utilization reported for multiple ONN size for Xilinx 7-series FPGA.

**#Neurons**	**#Synapses**	**LUTs (%)**	**Flip-Flops (%)**
15	225	900 (1.7)	721 (0.68)
60	3,600	6,300 (12)	2,756 (2.59)
100	10,000	30,033 (56)	4,985 (5)
120	14,400	38,372 (72)	5,970 (6)
140	19,600	46,900 (88)	6,955 (7)
150	22,500	65,251 (123)	7,447 (7)

These first simulation results confirm the digital ONN capability to perform pattern recognition.

#### 3.1.2. FPGA Implementation

We validate and characterize our digital ONN implementation by reporting on ONN pattern recognition accuracy (error rate) for multiple parameters. First, we use a low frequency and we perform the same tests as in simulation to check the ONN FPGA implementation. Then, we compare the error rate for multiple training configurations. Next, we observe the error rate for faster frequencies to check the frequency limit. Finally, we measure the computation time and calculate the number of FPS treatable by the ONN.

##### 3.1.2.1. ONN Training Configuration

Here, we present the ONN operation for multiple training configurations. Results are shown in Table 4 for the 5 × 3 ONN and the 10 × 6 ONN. First, we validate the ONN implementation by comparing results with simulation tests. Implementation tests with the same training configuration as simulation tests give equal results. Then, we observe that the error rate increases with the number of stored patterns for both learning rules. With the 5 × 3 ONN, we obtain similar results with both Hebbian and Storkey learning rules. However, with the 10 × 6 ONN, we obtain significant error rate differences. We notice that weights trained with Storkey give a lower error rate than weights trained with Hebbian for four pattern combinations out of five. Observations also indicate which training configuration can be the best option for a particular application considering an acceptable error rate. If we consider 0% acceptable error rate for the 10 × 6 ONN, we can store patterns from 0 to 3 with the Hebbian learning rule, and we can add digit 4 if we use the Storkey learning rule.

**Table 4:** Experimental error rate of the 5 × 3 and the 10 × 6 ONN implemented on FPGA for various training configurations.

**ONN**	**Stored**	**Learning rule**	**Test**	**Errors**	**Error rate**
	**patterns**		**images**		**(%)**
5 × 3	0,1	Hebbian	10	0	0
		Storkey	10	0	0
5 × 3	0,2	Hebbian	10	0	0
		Storkey	10	0	0
5 × 3	1,2	Hebbian	10	0	0
		Storkey	10	0	0
5 × 3	0,1,2	Hebbian	15	1	6.67
		Storkey	15	1	6.67
10 × 6	0,1,2,3	Hebbian	20	0	0
		Storkey	20	0	0
10 × 6	0,1,2,3,4	Hebbian	25	5	20
		Storkey	25	0	0
10 × 6	0,1,2,3,4,5	Hebbian	30	9	30
		Storkey	30	1	3
10 × 6	0,1,2,3,4,5,6	Hebbian	35	21	60
		Storkey	35	4	11
10 × 6	0,1,2,3,4,5,6,7	Hebbian	40	35	87.5
		Storkey	40	4	10

##### 3.1.2.2. ONN Frequency

**Figure 8:**
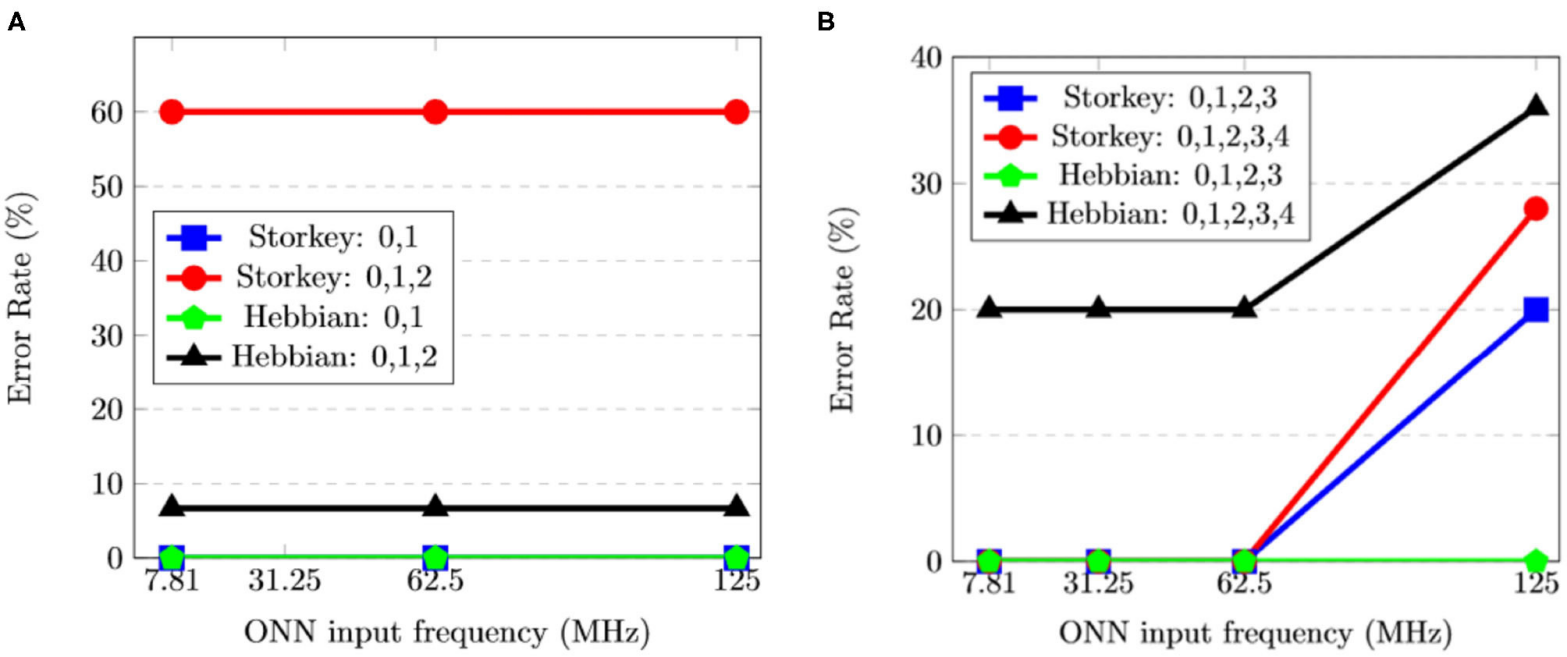
Experimental results of the error rate (%) for various frequencies (MHz) and training configurations (learning rule and pattern combination) for **(A)** the 5 × 3 ONN, and **(B)** the 10 × 6 ONN.

Simulation reveals ONN maximum frequency depends on the applied learning rule. With FPGA implementation, ONN input frequency choice is limited. We perform FPGA implementation experiments on the same range of frequencies to check if implementation and simulation results match. Experiments on 5 × 3 ONN perform similarly at 7.8125, 62.5, and 125 MHz as shown in Figure 8A. Thus, ONN can run a given test set at higher frequencies than the evaluated limit (83, 33 MHz) for all tested training configurations. The difference in frequencies can be explained by the way they are measured—in simulation, ONN frequency was evaluated with global static timing analysis, whereas in experiments, frequency is evaluated on a specific test set. Figure 8B shows the 10 × 6 ONN error rate at different frequencies for four training configurations. We observe a trade-off between error rate, operating frequency, and training configuration. Also, we note that for each training configuration, only a frequency of 125 MHz impacts the 10 × 6 ONN error rate.

Considering simulation and implementation results, we assess the 5 × 3 ONN on FPGA maximum operating frequency to be 62.5 MHz, and the 10 × 6 ONN on FPGA maximum operating frequency to be 31.25 MHz. In our next experiments, related to time measurements, we define a common digital ONN input frequency for 5 × 3 and 10 × 6 ONNs. We set it to 31.25 MHz as it is the highest common operating frequency.

##### 3.1.2.3. ONN Computation Time

We assess the computation speed of our ONN implementation with time measurements. We experimentally measure the ONN initialization time needed to apply the input image to ONN, and the computation time, from the end of the initialization process to the *steady* signal's activation time (see section 2.3). We measure ONN timings for multiple training configurations shown in Table 5. We choose training configurations that showed a 0% error rate to avoid the maximum computation time clamped to 160 μ*s* when ONN does not converge. We measure the computation time as the time required for the ONN to reach a stable correct output pattern (0% error rate). We set the digital ONN input frequency to 31.25 MHz.

**Table 5:** ONN timing performances for various sizes (number of neurons) and learning rules.

**Learning**	**ONN**	**Stored**	**Initialization**	**Computation**	**FPS**
**rule**		**patterns**	**time**	**time, avg**	
			**(us)**	**(us)**	
Hebbian	5 × 3	[0,1]	2	5.04	142,045
Hebbian	10 × 6	[0,1,2,3]	7.8	5.2	76,923
Storkey	5 × 3	[0,1]	2	5.05	141,844
Storkey	10 × 6	[0,1,2,3]	7.8	5.4	75,757

Computation time, initialization time, and FPS results are listed in Table 5. We initialize the ONN by presenting data serially to the ONN, so it depends on the size of the ONN, regardless of the applied learning rule. It explains why the initialization time is longer for 10 × 6 than 5 × 3 ONN.

We observe that ONN computation time does not vary and slightly increases with ONN size. It is an attractive feature of the ONN concept in which convergence is achieved in a few oscillation cycles independently of the number of neurons. It is also worth mentioning that the degradation of FPS performance is due to the initialization time that grows linearly with the number of neurons because of its serial implementation. It could be mitigated by using a different initialization approach.

Simulation and implementation characterizations allowed us to validate our ONN digital design to perform pattern recognition with a minimum error rate of 0%. We performed multiple experiments to highlight the advantages and limitations of the digital ONN. The main limit concerns the digital ONN FPGA resources (LUTs, Flip-Flops). The current digital ONN design implemented on the *XC*7*Z*020 − 1*CLG*400*C* FPGA is limited to a 100 neurons. In contrast, we highlighted the short-time computation required by the digital ONN. We can compute each pattern with an average of 5 μ *s* at 31.25*MHz* frequency for both 5 × 3 and 10 × 6 ONNs. So, ONN size does not impact the computation time, which is an important feature of ONN to further explore design methods to upscale its size.

### 3.2. Digits Recognition Application Results

**Figure 9:**
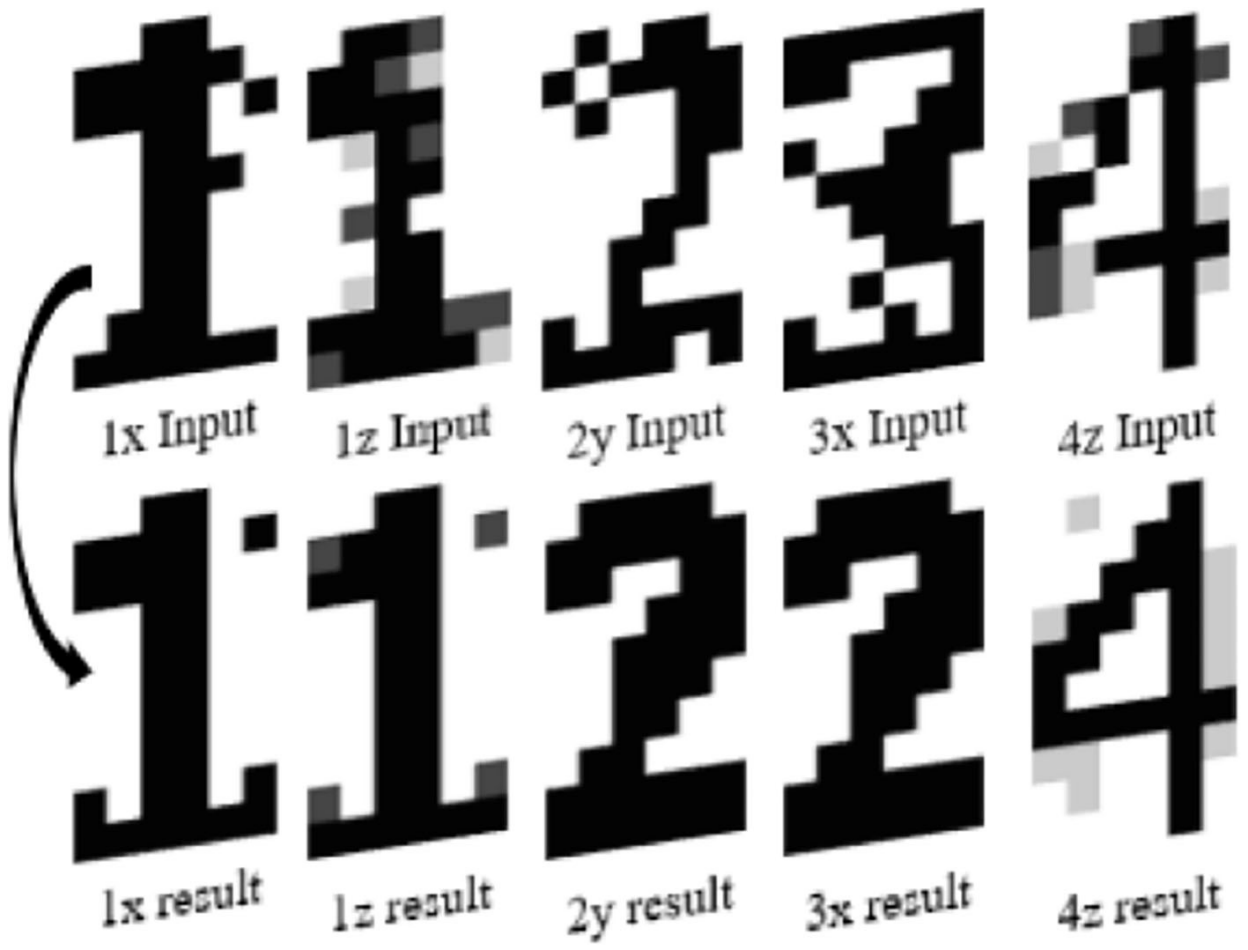
Representation of error images for 10 × 6 ONN digit recognition application showing incorrect pixels.

We validate the image recognition application by comparing errors with previous characterization tests. We use the output HDMI screen to identify recognized images and errors of the digital ONN image recognition application. We expect results to match with characterization ones and as expected, we find five images not correctly recognized, see Figure 9. Output patterns displayed on the screen reveal that for each not-correctly recognized image, ONN is close to a correct reference pattern with only a few pixels wrong. However, the reference pattern is not necessarily the expected one, as for the image 3x. We expected a 3, but the result is closer to a 2. Some errors can be explained by test images that correspond to corrupted digits too far in their HD from reference patterns. With this application, we demonstrate the feasibility of using an ONN inside a complete design. Also, we reach 20% error rate with this application using Hebbian weights, but we know thanks to characterization, that Storkey weights can reach 0% error rate. Besides, we know that 10 × 6 ONN takes 7.8 μ*s* to be initialized, 5 μ*s* to stabilize on average, and 160 μ*s* if it does not stabilize. As the camera provides an image every 15*ms*, the 10 × 6 ONN does not create any latency for the image recognition application, so it respects real-time requirements. It validates our digital ONN design as a solution for image recognition applications and encourages us to look into other embedded applications.

## 4. Discussion

Our work presents the design and performances of a novel fully digital ONN implementation. Further, we demonstrate ONN on FPGA implementation for image recognition applications. It is a proof-of-concept on the potential of ONN as an alternative computing paradigm. Here, we present the digital ONN design aspects and their advantages, limits, and future directions.

### 4.1. Advantages

Presently, we developed ONN designs that exhibit good performances with 5 × 3 and 10 × 6 ONNs.

An interesting feature of our approach in comparison with Jackson et al. (2019) is that by resorting to the 1-bit oscillation at the neuron's output, multipliers are avoided in the synapses block, while still retaining a multi-level neuron. This is possible because we encode the state in the neuron's oscillation phase.

Another advantage of our approach is the easiness of training. Results reported in this paper have been obtained using simple Hebbian and Storkey rules. Given the patterns to be stored/recognized, weights are calculated offline by using matrix operations, while training other neural models can be a very time-consuming operation. Limited retrieval capacity has been obtained in some of the experiments described. However, the accuracy of the ONN can be increased by resorting to enhanced learning rules.

Finally, a fundamental advantage of ONN is the fast computation. ONN parallel behavior allows the independence of the computation time and the network size. In this paper, we achieved more than 70000 FPS with 10 × 6 ONN and a serial initialization of the neurons.

### 4.2. Limitations and Future Directions

The present digital ONN design has also limitations.

First, our ONN is a preliminary design developed to validate the concept, and different optimization procedures at different levels are currently being carried out.

Second, the limited FPGA resources introduce some constraints on the ONN size that can be implemented on FPGA (see Table 3). Such limited resources are the computation resources (LUTs). They are used to implement the combinatorial synapses block whose size increases quadratically with the number of neurons.

The limited size does not allow for comparison with standard benchmark sets usually used to evaluate neural networks. For example, comparing ONN with SNN is not trivial because of the paradigm differences, such as the different network architectures and learning algorithms. To make a meaningful comparison, a common ground is necessary with a common application and benchmark. Comparison to other reported similar FPGA-based implementations of neural networks is meaningful only if the same ONN size are developed.

The Associative Memory Neural Networks (AMNNs), such as HNNs, are the closest ANNs that can be compared with ONNs. In the existing literature, we found digital implementations of AMNNs such as Leiner et al. (2008), Mansour et al. (2011), and De Abreu de Sousa et al. (2014). The most relevant comparisons can be made with the digital design from De Abreu de Sousa et al. (2014)'s work, which is also the most recent one. In this work, authors perform a frequency study for multiple HNN sizes, from 16 neurons to 32 neurons. Stored patterns resemble our stored patterns representing digits. For example, they use 8 × 4 representations of the letter U and the number 5. Tests are also similar as they use stored and corrupted patterns as inputs.

Table 6 shows the comparison between (De Abreu de Sousa et al., 2014)'s HNN and our ONN. Results show that ONN has a higher operating frequency than HNN. Such as in the 32 neurons case, the maximum HNN frequency is 33.55 MHz, but ONN can run faster, up to 60.61 MHz for 10 × 6 ONN. We do not have information about the computation time for the 32-neuron HNN, however, if we compare the 16-neuron HNN with the 5 × 3 ONN, we expect similar trends in frequencies and computation times. Though it is difficult to make any conclusive comparisons, it seems that our larger ONN can operate at higher frequencies than the cited HNN.

**Table 6:** Frequency, computation time and resource comparison of ONN and HNN designs.

**Neural**	**Size**	**Frequency**	**Computation**	**Computation time**	**Resources**
**network**		**(MHz)**	**time (us)**	**(clock cycles)**	**(LUTs)**
			**Max - Avg**	**Max - Avg**	
HNN (*)	16	81.39	x - 1.3	x - 100	390
HNN (*)	32	33.55	x - x	x - x	700
ONN					
- Hebbian	15	83.33	2.74 - 0.92	228 - 77	958
- Storkey	15	83.33	1.18 - 0.77	98 - 64	800
ONN					
- Hebbian	60	64.1	3.53 - 1.44	226 - 92	6,426
- Storkey	60	60.61	1.75 - 1.18	106 - 72	6,192

(*) Refers to De Abreu de Sousa et al. (2014). Note, HNN resource estimation comes from data extraced from De Abreu de Sousa et al. (2014) and FPGA documentation. Also note, HNNs are implemented on a Xilinx 3-series FPGA (4-input LUTs) while ONNs are implemented on a Xilinx 7-series FPGA (6-input LUTs).

Motivated by the potential of the proposed ONN, we are currently exploring optimizations in several directions like hardware resources, frequency, and accuracy. For example, using a faster internal clock than the oscillator frequency, a sequential implementation could be used to reduce the size of this resource-consuming block. Also, we can explore additional learning rules to increase accuracy. At this time, we have only studied Hebbian and Storkey, which are local and incremental with a limited storage capacity, but other learning rules will also be explored to have a better assessment of learning rules suitable for ONNs. For example, the pseudo-inverse rule (also called projection rule) can increase HNNs storage capacity and improve accuracy (Wu et al., 2012; Sahoo et al., 2016), but is not local nor incremental. Moreover, recent works have explored learning rules with self-feedback connections (non-0 diagonal), and have shown higher accuracy for a high number of stored patterns (Liou and Yuan, 1999; Folli et al., 2017; Rocchi et al., 2017; Gosti et al., 2019). In summary, despite the present limitations of the ONN, features in terms of FPS, computation time and training, are encouraging toward the exploration of a wider range of applications.

## 5. Conclusion

In this paper, we carried out the questions—can we use ONN for image recognition, and—what are the advantages and limitations of ONN for AI at-the-edge applications. To do so, we presented a proof of concept of the ONN neuromorphic computing paradigm with a fully digital design. We validated the computing capability of a 5 × 3 ONN and a 10 × 6 ONN performing pattern recognition both in simulation and FPGA implementation. We used Hebbian or Storkey learning rules to train our ONN. For both learning rules, with three stored patterns, the 5 × 3 ONN retrieved 14 test patterns out of a test set of 15. For the 10 × 6 ONN with five stored patterns, results differ from Hebbian and Storkey learning rules. ONN with weights computed with Storkey can retrieve 25 test patterns out of 25, but using Hebbian ONN can only retrieve 20 test patterns out of 25. Further experiments confirmed that Storkey is more accurate than Hebbian for an equal number of stored patterns. We performed additional experiments to characterize our digital ONN. First, we estimated by simulation a maximum operating frequency for the 5 × 3 ONN to 83,33 MHz. Then, we showed that for a specific application, the ONN implemented on FPGA could go up to 125 MHz, without any changes on the ONN operation. Besides, we performed a timing analysis on digital ONNs. We measured the initialization time needed to apply the input pattern to the ONN, and the computation time, needed by the ONN to stabilize to a stored pattern. From measurements, we were able to calculate the maximum FPS (Frames per second). For the 10 × 6 ONN, we obtained a maximum FPS around 76000, at 31.25 MHz with a training configuration resulting in all test patterns successfully retrieved. Then, we embedded the 10 × 6 ONN into a complete image recognition application performing digit recognition from a camera stream. It respected real-time constraints, and we demonstrated that the ONN paradigm can fit with AI at-the-edge image recognition application. Thus, despite the size limitation (about a 100 neurons) of our digital design due to high FPGA resource consumption, the huge potentiality of ONN is undeniable. ONNs are still in their infancy for comparison with standard benchmarks and it is the focus of future works. The potential of the proposed ONN propels further investigation to explore its capabilities on diverse applications for AI at-the-edge.

## Data Availability Statement

The raw data supporting the conclusions of this article will be made available by the authors, without undue reservation.

## Author Contributions

AT-S motivated the project and experiments. MJ, JN, and MJA conducted the digital ONN design development and simulation characterization. MA and TGi developed the ONN design implementation on FPGA, image recognition application, and performed all measurements. BL-B, TGo, and TH were involved in the discussion and editing of the manuscript and provided valuable inputs at multiple stages of this work. All authors contributed to the article and approved the submitted version.

## Conflict of Interest

TGo and TH were employed by the company A.I. Mergence, Paris. The remaining authors declare that the research was conducted in the absence of any commercial or financial relationships that could be construed as a potential conflict of interest.

## Publisher's Note

All claims expressed in this article are solely those of the authors and do not necessarily represent those of their affiliated organizations, or those of the publisher, the editors and the reviewers. Any product that may be evaluated in this article, or claim that may be made by its manufacturer, is not guaranteed or endorsed by the publisher.
